# Advances in Pathogenesis of Sjögren's Syndrome

**DOI:** 10.1155/2021/5928232

**Published:** 2021-10-07

**Authors:** Yao Tian, Hongyi Yang, Na Liu, Yan Li, Jie Chen

**Affiliations:** ^1^Department of Rheumatology and Clinical Immunology, Jiangxi Provincial People's Hospital Affiliated to Nanchang University, Nanchang, China; ^2^Department of Rheumatology and Clinical Immunology, The First Affiliated Hospital of Xiamen University, Xiamen, China; ^3^Department of Science and Techonology, Jiangxi Provincial People's Hospital Affiliated to Nanchang University, Nanchang, China

## Abstract

Sjögren's syndrome (SS) is a chronic autoimmune disease of unknown etiology that mainly involves exocrine glands. Patients present with dry mouth and eyes, fever, arthralgia, and other systemic symptoms. In severe cases, the quality of life of patients is affected. At present, there is no cure for SS, and the treatment options are extremely limited. In recent years, studies of patients and animal models have identified abnormalities of immune cell function and cytokines to be involved in SS. A systematic review of the literature may clarify the etiology and pathogenesis of SS, as well as provide a theoretical basis for the development of new drugs for the treatment of SS.

## 1. Introduction

Sjögren's syndrome (SS) is a chronic autoimmune disease of unknown etiology that involves exocrine glands. SS patients present with dry mouth and eyes, fever, joint pain, and neurological symptoms. In severe cases, the quality of life of patients is compromised, thereby predisposing them to mental illnesses, such as depression [1, 2]. SS can be divided into primary SS (pSS) and secondary SS (sSS). An absence of diseases other than rheumatologic disease characterizes pSS [3, 4], whereas sSS occurs secondary to autoimmune diseases, such as systemic lupus erythematosus, systemic sclerosis, or rheumatoid arthritis. The average incidence of SS is 6.0 per 100,000 people and is 10-fold higher in women compared to men. The incidence of SS increases with age; the highest incidence in women occurs at 55–64 years and in men at 65–74 years [5]. Many researchers have extensively studied the causes underlying SS and have found a multifactorial pathogenesis of SS, including environmental, genetic, neuroendocrine, and immune (related to immune cells and cytokines) factors [1]. The pathogenesis of SS has not been fully elucidated, and there remain difficulties in the clinical diagnosis of SS. This article summarizes the recent advances in pathogenesis, evidence from animal models, possible causes, and drug treatments of SS.

## 2. Genetic Susceptibility

Genetic susceptibility plays an important role in the pathogenesis of SS. Previous studies used SS genome-wide association analysis to identify IRF5-TNPO3, STAT4, IL12A, FAM167A-BLK, DDX6-CXCR5, and TNIP1 as risk sites and IRF5 and STAT4 as susceptibility genes for SS [6]. IRF5 is a transcription factor that acts downstream of Toll-like receptor (TLR) and type I interferon (IFN) receptor and promotes the expression of various proinflammatory factors, whereas STAT4 is a transcription factor involved in the production of IFN and causes autoimmune abnormalities [7–9]. As mentioned previously, the incidence of SS is significantly higher in women than men. A study of 242 female patients with pSS found that GTF2I and RBMS3 were the two most important susceptibility genes for pSS in women [10], which explains the high incidence of pSS in women. Long noncoding RNA (lncRNA) does not code for functional proteins, rather regulates gene expression through effects on the chromosomes, DNA, transcription, and posttranscriptional modification. lncRNA also regulates the differentiation and development of immune cells and the production of inflammatory mediators, thereby affecting the development of autoimmunity [11]. Many lncRNAs are dysregulated in pSS and may be responsible for the increased risk of SS [12]. The role of microRNAs (miRNAs) in the pathogenesis of autoimmune diseases has recently received a large amount of attention; miRNAs participate in various physiological and pathological processes, such as cell metabolism, differentiation, proliferation, and apoptosis, as well as in immune responses [13]. The expression levels of miRNA-181a and miRNA-16 related to pSS are reduced in the salivary glands of pSS patients [14]. There is an increased expression of miRNA-146a and a decreased expression of miRNA-155 in the peripheral blood mononuclear cells of pSS patients. The levels of expression of these miRNAs correlate with the patient's clinical manifestations [15]. These data indicate that miRNA-181a, miRNA-16, miRNA-146a, and miRNA-155 play a role in the pathogenesis of pSS. In addition, epigenetics, such as DNA methylation and histone modification, may be related to the occurrence of SS [16, 17].

## 3. Infection

Infection plays a critical role in the development of many autoimmune diseases [18]. Viral infection is a potential risk factor for SS [19, 20]. The presence of viruses can alter the biology of epithelial cells, which may lead to overexpression of type I IFN-induced genes, inflammation, and tissue damage. Many viral proteins share 5–6 consecutive amino acid residues with Ro-60 antigen, and this sequence similarity may be responsible for generating autoimmune responses [21, 22]. Epstein-Barr virus infection can prolong the B cells and lead to their abnormal activation in SS patients [23]. The human T lymphotropic virus type 1 (HTLV-1) can infect T cells, B cells, and bone marrow cells, leading to cell activation and proliferation. Studies have found that the salivary glands of HTLV-1 patients demonstrate lymphocyte infiltration and inflammation, and these patients may develop symptoms similar to SS [24]. In addition, cytomegalovirus, human herpesvirus type 8, and hepatitis C virus may also be closely related to the development of SS [25].

## 4. Salivary Gland Epithelial Cells

The salivary gland epithelial cells (SGECs) in SS are key to the initiation and propagation of the immune response. Abnormal exocrine gland homeostasis is considered to be the initial step in the development of SS. Exocrine dysfunction occurs before inflammation [26, 27]. After stimulation by antigens, SGECs secrete proinflammatory factors that cause infiltration by immune cells and act as antigen-presenting cells to induce differentiation of T lymphocytes [28–31]. CD80/86 is expressed on the surface of SGECs in SS patients. Treatment of the NFS/sld mouse SS model with anti-CD86 antibody improves the autoimmune damage to salivary and lacrimal glands, indicating that these epithelial cells may have antigen-presenting functions and may increase T cell proliferation and activation, resulting in damage to the glands [32] (Table 1). Studies have found that SGECs in SS patients promote the activation of naive B cells in peripheral blood, which confirms that SGECs mediate the activation and differentiation of B cells in SS [33]. TLRs, an important class of protein molecules, are involved in innate immunity. These receptors are expressed on immune cells and some epithelial cells. Binding of TLRs to the receptor induces the production of type 1 IFN and other cytokines. In addition, there is an increased expression of TLRs on the SGECs in SS. These results indicate the role of SGECs in the pathogenesis of pSS [34]. TLR8ko mice show salivary adenitis, cytokines, and anti-SSA and anti-SSB autoantibodies, which were not present in TLR7/8KO mice, suggesting that these changes depend on TLR7, further confirming the important role of TLR7 signaling in the local and systemic manifestations of SS [35] (Table 1).

## 5. B Cells

B cells play an important role in the development of SS. Overactivation of B cells in the exocrine glands of SS patients manifests as gland swelling and the production of anti-SSA and anti-SSB autoantibodies [33]. C3H/HeJ mice immunized with Ro60-316-335 peptide spontaneously develop lacrimal gland inflammation and a variety of serum autoantibodies (Table 1). Damage to the mouse exocrine glands was reduced after treatment with CD20 monoclonal antibody-depleted B cells compared to the control group [21]. In line with this, CD20 monoclonal antibodies are used clinically to treat SS. For example, rituximab, a chimeric antibody against CD20 antigen, depletes B cells and reduces B cell-derived proinflammatory cytokines. Symptoms of SS significantly improve with rituximab treatment. The aforementioned evidence supports the important role of B cells in the pathogenesis of SS [36]. Ianalumab (VAY736) is a monoclonal antibody that depletes B cells and blocks the B cell activator receptor. It improves the symptoms of pSS, without causing significant side effects [37]. The C57BL/6.NOD-Aec1Aec2 mouse model of SS demonstrates increased specific chemokines, upregulation of chemokine receptors in the salivary glands, and infiltration of perivascular B lymphocytes, suggesting that B lymphocytes are crucial for the development of SS [38] (Table 1). Studies have evaluated the expression of B cell marker CXCL13 and found that CXCL13 is elevated in a variety of autoimmune diseases, including SS [39, 40].

## 6. T Cells

Infiltration by a large number of T cells and the expression of various cytokines in the exocrine glands of SS animal models revealed that T cells and related cytokines play an important role in the development of SS [41]. Kurosawa et al. [42] found increased expression of CXCR4 and CXC12 in the aly/aly spontaneous SS mouse model, which resulted in abnormal infiltration by a large number of T cells (Table 1). Specific nuclear matrix binding domain binding protein 1 (SATB1) is specifically expressed in the T cells of mature hematopoietic cells, and SATB1 gene-specific knockout (SATB1cKO) mice have spontaneously reduced salivary secretion. Additionally, a large number of T cells, as well as serum anti-SSB and anti-SSA autoantibodies, are present in the salivary glands of SATB1cKO mice (Table 1). RAG2^−/−^ mice that received SATB1cKO mouse T cells (mice with RAG2 gene deletion cannot produce mature lymphocytes) had reduced salivary secretion and damaged salivary acinar cells, which suggest that T cells play an important role in the pathogenesis of SS [43].

Th17 cells are involved in a variety of autoimmune diseases, and the nuclear receptor retinoic acid-related orphan receptor (ROR*γ*t) is an essential transcription factor for the differentiation of Th17 cells. Salivary glands of ROR*γ*t transgenic (ROR*γ*t Tg) mice were stained with hematoxylin and eosin, which revealed sialadenitis due to excessive CD4^+^ T cell infiltration (Table 1). The infiltration of CD4^+^ T cells and cytokines in the salivary gland was significantly increased compared to the control group [41]. These results provide strong evidence for the involvement of ROR*γ*t and CD4^+^ T cell subsets in the pathogenesis of SS. Some studies used salivary gland protein to immunize wild-type mice and IL-17KO mice to establish SS mouse models. Wild-type mice immunized with salivary gland protein demonstrate SS symptoms; Th17 cells in the salivary glands increased. IL-17KO mice immunized with salivary gland protein did not exhibit SS symptoms or histopathological changes. Th17 cells produced *in vitro* were adoptively transferred to IL-17KO mice, which resulted in reduced salivary secretion and increased levels of serum autoantibodies [44] (Table 1). Therefore, Th17 cells are involved in the pathogenesis of SS.

Treg cells negatively regulate the immune processes [45]. Studies have shown that although the exocrine glands of SS mouse models are infiltrated with Treg cells, SS-like symptoms (such as salivitis) still occur, which suggests that the number of Treg cells may not be sufficient to inhibit effector T cell-induced thymectomy in mice [46]. A recent study observed a large number of CD8^+^ T cells in the exocrine glands of SS animal models and patients; removal of these resident CD8^+^ T cells after disease onset can alleviate the pathology [47], which may form the basis of future SS treatments.

## 7. Dendritic Cells

Dendritic cells (DCs) are essential for initiating and maintaining immune responses. DCs are the most important antigen-presenting cell in the body. These cells phagocytose antigens and present them to T lymphocytes [48]. Studies have found that a decreased number of DCs in the peripheral blood of pSS patients occurs due to the migration of DCs from peripheral blood to the secretory glands, which leads to the differentiation of Th1 cells in the salivary glands and the production of a large amount of IFN-*γ* that induces inflammation [49]. Results of studies of the NOD mouse model (Table 1) support the following hypothesis: the early NOD mouse model demonstrates the accumulation of DCs in the submandibular gland, which stimulates initial T cells to initiate autoimmunity, resulting in the infiltration of T cells in the submandibular gland [50]. DCs promote the activation of initial T cells, thereby producing proinflammatory cytokines and stimulating the infiltration of T cells in the salivary glands, which causes sialadenitis and the pathogenic changes of SS.

## 8. Cytokines

Cytokines are small molecular proteins with biological activity. They are synthesized and secreted by nonimmune cells (endothelial cells, epidermal cells, and fibroblasts) and immune cells (monocytes, macrophages, T cells, B cells, and natural killer cells). Current studies have shown that the combined action of various cytokines leads to the development of SS and promotes inflammation [51].

### 8.1. Interleukins

Interleukins are a class of cytokines produced and used by a variety of cells. IL-1 cytokines are key members of the IL-1 family. Studies on autoimmune regulation (Aire) deficient mice have shown that IL-1 receptor knockout reduces ocular surface keratosis but not lymphocyte infiltration in lacteal glands; therefore, IL-1 receptor-targeted therapy may only improve ocular symptoms [52] (Table 2). IL-33 is also a member of the IL-1 family and has dual functions as a nuclear factor and a cytokine. In SS patients, the serum level of IL-33 is significantly increased to promote IFN-*γ* and inflammation, which in turn increases the activation of the IL-33/ST2 pathway, leading to worsening of the disease [53].

IL-2 and IL-21 belong to the IL-2 family. Some studies have found that treatment with low-dose IL-2 increases the number of Treg cells compared to the baseline, decreases the Th17/Treg ratio, and improves the symptoms of SS. These results indicate that IL-2 promotes the proliferation of Treg cells, suggesting that more attention should be paid to immune regulation, rather than immunosuppression, for the treatment of SS [54]. Act1 attenuates IL-21-induced activation of STAT3, thereby inhibiting B cell activity. Mice lacking ACT1 exhibit SS-like symptoms [55] (Table 2). It also affects IFN-1 signaling and plays an important role in the pathogenesis of SS [56, 57].

The IL-17 family includes IL-17 and IL-25 (IL-17E). Early studies reported that IL-17 produced by Th17 contributes to the development of SS [58, 59]. However, later studies found that IL-17-deficient ROR*γ*t-Tg mice also spontaneously develop salivitis, which suggests that IL-17 is not necessary for the development of salivitis in ROR*γ*t-Tg mice [41]. IL-25, also known as IL-17E, belongs to the IL-17 family of cytokines. IL-25 has dual immunoregulatory roles: it upregulates the immune response mediated by Th2 cells and downregulates the immune response mediated by Th1 and Th17 cells [60]. IL-25 inhibits the differentiation of CD4+ T cells into Th17 cells, thereby inhibiting inflammation [61]. IL-12 and IL-23 also play an important role in the development of autoimmune processes [62, 63].

IL-22 belongs to the IL-10 family, and IL-22 levels in the salivary glands and serum of SS patients are significantly increased. IL-22 is associated with insufficient salivary secretion in SS patients, suggesting that it may be a potential therapeutic target [63].

IL-14 is a cytokine that is also known as B cell growth factor. Studies of transgenic mice with IL-14*α* suggest that IL-14 may play a role in the development of autoimmunity by regulating B cell function [64] (Table 2).

### 8.2. TNF Family

The tumor necrosis factor (TNF) family refers to a group of cytokines that cause cell death (apoptosis). TNF-*α* is a key contributor to SS pathogenesis and is secreted by CD4^+^ T cells, monocytes, and epithelial cells. In addition, overexpression of TNF-*α* induces symptoms of exocrine adenitis in mice, without serum autoantibodies [65] (Table 2). Therefore, the relationship between TNF-*α* and development of SS remains unclear. Two recent clinical trials of etanercept, a TNF-*α* antagonist, found no evidence of benefit for SS patients [66, 67]. Larger trials may be required to determine the efficacy of etanercept in the treatment of SS.

B cell-activating factor (BAFF) is a member of the TNF superfamily that regulates immune responses. BAFF is a cytokine with important effects on the development and selection of B cells. A proliferation-inducing ligand (APRIL) is homologous to BAFF and is abnormally increased in the serum and inflammatory labial gland tissue of pSS patients, which indicates that APRIL participates in the pathogenesis of pSS by stimulating the proliferation of B cells [68]. Sjöstrand et al. [68] found increased BAFF expression in the immune cells of pSS patients, especially neutrophils. In addition, they identified a highly conserved IFN-stimulated response element (ISRE) site near the BAFF gene promoter, which was functionally verified. IRFs combined with this site could regulate BAFF expression, production of B cells, and production of a variety of autoantibodies, leading to the development of autoimmune diseases, such as pSS. Additionally, varying degrees of acinar destruction and a high degree of lymphocyte infiltration were observed in the saliva and submandibular glands of BAFF Tg mice [69] (Table 2). Several drugs act on BAFF. Belimumab is a fully humanized monoclonal antibody against soluble BAFF, which is used for treating SS patients [70].

### 8.3. Interferon

IFN is a protein produced from different cell types and has powerful immunomodulatory effects. There are three types of IFNs: I, II, and III. IFN-*α* and IFN-*β* are the most widely studied type I IFNs in SS. Some studies have found type I IFN-induced gene overexpression in the salivary glands and peripheral blood of SS patients, as well as increased levels of type I IFN, suggesting that type I IFN is a key factor in the pathogenesis of SS [71].

IFN binds to its receptor and activates the JAK/STAT pathway. JAK inhibitors can treat pSS by downregulating the STAT pathway [72]. The mechanism of action of JAK inhibitors includes the regulation of pSTAT-1Y701, pSTAT-3Y705, and activated STAT-3 proteins induced by IFN-*α* and IFN-*γ* in human SGECs. Studies have found increased type I IFN levels and type I IFN-induced gene overexpression in the salivary glands and peripheral blood of pSS patients, suggesting that the type I IFN pathway plays an important role in the pathogenesis of pSS [71]. Therefore, JAK inhibitors (such as tofatinib and baritinib) that act downstream of IFN may be used to treat SS. The results of a prospective, randomized, double-blind, multicenter clinical trial showed that tofacitinib improves dry eyes in SS patients. Baritinib inhibits JAK/STAT signal transduction, IFN-*γ*-induced CXCL10 expression, and immune cell chemotactic activity, indicating its effectiveness for the treatment of SS [73, 74]. Circulating IFN-*α* was significantly reduced in SS patients compared to healthy subjects [75]. Several clinical studies have shown a significant increase in salivary production in SS patients treated with human IFN-*α* for 24 weeks [76, 77]. However, the mechanism of IFN-*α* production is not clear, but it may be due to the upregulation of aquaporin 5.

### 8.4. Osteopontin

Osteopontin (OPN) is a multifunctional cytokine with diverse sources of production [78]. An SS-like phenotype appears in OPN transgenic mice, which suggests that OPN is related to the etiology of SS [79] (Table 2).

## 9. Neuroendocrine

The incidence of SS is significantly higher in women than in men, suggesting that the hypothalamic-pituitary-gonad axis plays an important role in the pathogenesis of SS. In women, pSS onset usually occurs after menopause, probably because of insufficient estrogen secretion. In animal models, sex hormones affect humoral immunity and cellular immune response [80]. Knockout of the mouse aromatase gene (ArKO mice) results in a lack of estrogen. These mice spontaneously develop an SS-like phenotype, characterized by B cell infiltration in the salivary glands, destruction of acinar cells, and secretion of a variety of autoantibodies [81]. Aromatase deficiency causes M1 macrophages to produce proinflammatory cytokines in the adipose tissue and target organs, leading to SS-like lesions [82]. These findings suggest that estrogen plays a key role in the pathogenesis of SS. Wang et al. found that TLR8ko female mice had more severe salivary adeninflammation compared to male mice. TLR7 expression was almost twice as high in the salivary glands of TLR8ko female mice compared to male mice. TLR7 gene is located on the X chromosome, which may partly explain the sex differences in SS pathogenesis [40]. B cells play a key role in the pathogenesis of SS. Leptin, a type of adipokine, induces B cells to secrete a variety of proinflammatory cytokines [83]. It is controversial whether leptin plays a role in the development of SS. The distribution of leptin and its receptors is not altered in the salivary glands of SS patients. Lymphocytic infiltration of the salivary glands is not related to leptin or its receptors, indicating that they are not significant in the pathogenesis of pSS [84]. However, some studies of mouse models reported that the levels of serum leptin and its receptors in the salivary glands are related to the severity of SS [85]. Therefore, leptin may be related to the development of SS. The CD25 null mouse model is an animal model of SS that involves the eyes and lacrist glands. Stepp et al. showed that, compared with wild-type mice, the CD25 null SS mouse model demonstrated fewer nerves in the cornea and reduced corneal sensitivity, leading to ocular symptoms of SS [86]. This suggests that changes in the nervous system are also involved in the presentation of SS symptoms.

## 10. Conclusion

The pathogenesis of SS is unclear, and the treatment strategies are limited. SS animal models and patients share some common features, which help to understand the pathogenesis of human SS. Many studies have been conducted to explore the pathogenesis of SS. Various cells and cytokines are involved in SS, including SGECs, T cells, B cells, DCs, IFN, interleukin, TNF, and chemokines. Therefore, targeting these cells and cytokines may help to modulate the immune response and improve SS. B cells play a crucial role in the pathogenesis of SS; so, drugs targeting B cells play an important role in the treatment of SS. Most B cell therapies result in excessive consumption of B cells, which impairs immunity. Further studies are needed to determine the appropriate dosage of these drugs for SS treatment. SS is a complex disease, and SS treatment may involve signal transduction pathways, cytokines, cytokine receptor blockers, and antagonists. The establishment of an ideal animal SS model will allow the determination of SS etiology and discovery of potential therapeutic targets and methods for the prevention of complications. Such advancements will improve the prognosis and quality of life of SS patients.

## Figures and Tables

**Table 1:** Immune cells and Sjögren's syndrome mouse model.

Classification		Mouse model	Phenotype
Immune cells	Epithelial cells of salivary glands	Treatment of NFS/Sld mouse model with anti-CD86 antibody	Salivary adenitis, reduced lacrimal adenitis
		TLR8ko mice	Salivary adenitis, autoantibodies
	B lymphocytes	C3H/HeJ mice immunized with peptide Ro60-316-335	Lacrimal adenitis, autoantibodies
		C57BL/6.NOD-Aec1Aec2 mice	Salivary adenitis, lymphocytic infiltration
	T lymphocytes	Aly/Aly spontaneous mouse model	Lymphocytic infiltration
		STAB1ko mice	Reduced salivary secretion, infiltration of T lymphocytes in salivary glands, autoantibodies
		RoRrt transgenic mouse model	Salivary adenitis
		Th17-treated IL-17KO mouse model	Reduced salivary secretion, exocrine adenitis, autoantibodies
	Dendritic cells	NOD mouse model	DC infiltration of submandibular gland

**Table 2:** Cytokines and Sjögren's syndrome mouse model.

Classification			Mouse model	Phenotype
Cytokine	Interleukin	IL-1	Autoimmune regulation deficiency (Aire) mice with IL-1 receptor knockout	Decreased ocular surface keratosis
		IL-21	Mouse lacking Act1	Exocrine adenitis
		IL-14	Il-14*α* transgenic mice	Salivary adenitis, autoantibodies
	TNF	TNF-*α*	Overexpression of TNF-*α* in mice	Exocrine adenitis but no autoantibodies
		BAFF	BAFF transgenic mice	Exocrine adenitis, lymphocytic infiltration
	Osteopontin		Osteopontin transgenic mice	Exocrine adenitis

TNF: tumor necrosis factor; BAFF: B cell-activating factor.
